# Work Gains and Strains on Father Involvement: The Mediating Role of Parenting Styles

**DOI:** 10.3390/children10081357

**Published:** 2023-08-07

**Authors:** Eva Diniz, Lígia Monteiro, Manuela Veríssimo

**Affiliations:** 1 William James Center for Research, ISPA—Instituto Universitário, 1149-041 Lisboa, Portugal; ediniz@ispa.pt; 2 Centro de Investigação e Intervenção Social (CIS-IUL), Instituto Universitário de Lisboa (ISCTE-IUL), 1649-026 Lisboa, Portugal; ligia.monteiro@iscte-iul.pt

**Keywords:** father involvement, parenting, work–family conflict, parenting styles

## Abstract

The balance between work and family demands is one of the main challenges of contemporary parenting. However, most of the research has focused on mothers’ perspectives, with fathers’ perspectives about the links between work–family activities and father involvement, as well as the role of indirect effects, such as parenting styles, being less explored. This study aims to bridge these gaps by exploring whether work strains or gains are related to father involvement in childcare and the mediating role of parental styles, focusing on fathers’ reports. Working, married fathers of preschoolers (n = 411) self-reported about work strains and gains, parental styles, and father involvement. Structural equation modeling, using maximum-likelihood estimation, provided good fit indices. Results of the bootstrap analysis revealed how fathers’ gains indirectly increased involvement both in direct and indirect care through positive parental styles. Otherwise, fathers’ strains at work had a negative indirect effect on direct care through negative parental styles. Findings contribute to work–family interface by showing how parental styles account for mediating environmental challenges on father involvement.

## 1. Introduction

Demographic changes in families, namely due to both parents’ participation in a full-time job, introduced adjustments in parental roles and the need to conciliate parental and professional activities [1,2,3,4]. Contemporary parents accumulate work and parental demands, which is addressed as one of the major challenges of modern parenting [5,6], particularly during a child’s early years, given their greater dependence on parents [5,7]. The double demand for working and parenting is particularly relevant in Portugal, where 62% of children have both parents employed full-time, often for long working hours [6]. In addition, fathers have greater difficulty, in comparison to mothers, in accessing flexible work hours or family-friendly job policies, limiting their availability to daily caregiving [8], which has contributed to a higher rate of parent work–family conflicts in recent years [5,9].

Given that work demands may limit parents’ availability to perform parental activities, it is critical to understand the links between work–family conflict and father involvement, which translate to the multiple ways in which fathers may engage in childcare [3,4]. Work–family conflict conceptualizes the competing responsibilities between work and family demands [10]. However, the links between work–family conflict and father involvement may depend on the emotional climate in which parents raise their children, i.e., parenting styles [11]. Positive parenting styles translate parents’ ability to monitor a child’s behavior, addressing clear limit standards, whereas negative forms reveal strict discipline and control or, conversely, the lack of clear limit standards in a child’s interactions. Despite the fact parenting styles may play an important role in explaining the different associations between work–family demands and father involvement, their mediating role has been underlooked [4,12]. The current study relies on the complementary constructs of work–family strains and gains [13] to explore how work–family conflict influences father involvement and how parental styles mediate this association, focusing on fathers’ perspectives [3,8,14], moving beyond the dominant approach in mothers’ reports.

### 1.1. Work–Family Conflict and Parenting

The interdependence between professional and familial demands engenders work–family conflict. Originally, work–family conflict was conceptualized as the conflict resulting from pressures of both work and family tasks, often perceived as mutually incompatible given that the involvement with one task was perceived as an absence in the other one [10]. Accordingly, the accumulation of multiple and competing roles was perceived as compromising the participation in the family role, or vice versa, due to the individual’s limited time and energy, resulting in a negative interplay between work and family demands, i.e., work–family strains [8,13]. However, more recent approaches suggested that the conciliation of work and parental roles may be positive, resulting from the balance of costs and benefits of the involvement in multiple roles perceived as a source of pleasure and self-enrichment, i.e., work–family strains [5,15,16,17]. In this perspective, work activities become easier due to experiences, skills, and opportunities gained at home, or vice versa. When that happens, the relationship between work and family is enriching.

Work–family conflict is now perceived as continuous, with its strains and gains considered interdependent, influencing the quality of family life. Work–family gains are related to greater involvement with childcare, positive parenting behaviors, as well as positive parenting styles [15,17,18], whereas work–family strains are detrimental to parental quality and practices [7,19,20,21]. It is particularly relevant to examine the interplay between these variables in Portugal, which is a country in the European Union where more children (61%) have both parents working full-time jobs [22]. Portugal is also presented as a country with fewer family-friendly workplaces (e.g., allowing flexible work schedules), which may increase the challenges related to working and family demands [22,23]. However, it remains largely unexplored how parents face and manage work and family demands, not only related to their involvement with childcare activities but also to the quality of their parental roles [5,17,22,23].

### 1.2. The Interplay between Work–Family Conflict, Father Involvement, and Parenting Styles

Research on father involvement has moved forward in the examination of a father’s presence vs. absence to capture the diverse ways in which fathers may be involved with their children [2,3,4]. It is now expectable that fathers are involved in a wide range of childcare activities as “hands-on” nurturers and caregivers.

Father involvement is conceptualized as a broad concept merging the multiple ways in which fathers participate in their child’s life [3,4], such as caregiving activities, emotional support, discipline, and guidance [20,24,25,26]. Accordingly, father involvement may happen through direct interactions with the child (e.g., dressing, feeding, playing) or indirect ones (e.g., buying a child’s clothes and monitoring a child’s activities) [20]. Although engagement is considered a good indicator of an effective father’s direct participation in a child’s daily life [27,28], it is important to also consider the indirect aspects of care related to the responsibility for a child’s well-being, such as planning activities for the child or scheduling medical appointments, captured by the indirect care dimension [4,20].

Despite some studies in recent years uncovering how fathers tend to be more involved with overall domains of childcare [12,17,19,28,29,30], most of them do not discriminate specific forms of involvement, such as the disentangling of father involvement in caregiving from play/leisure activities. The studies that detail the dimensions of involvement reported how fathers are still typically more involved with child’s leisure/play activities rather than their caregiving [31,32,33,34]. However, the way in which fathers are involved with childcare is influenced by the context in which they are embedded, namely ecological and personal aspects [24,35]. Concerning ecological influences, the balance between work and family demands is considered crucial to family dynamics and well-being due to its spillover effect [36,37]. Considering personal influences, parenting styles are addressed as one important indicator of the father–child relationship quality [4,12], as displayed below.

Increased work–family strains have been related to lower involvement in childcare activities, such as preparing meals [38] or educational and leisure activities [39,40]. Also, greater working hours and more stressful work conditions are often related to greater work–family strains, which in turn is related to lower father involvement [3,41,42], namely in play and in the availability to be accessible to child requests [41,43]. Indeed, parents who perceive themselves as lacking the ability to manage work and family demands, i.e., work–family strains, revealed they have less involvement with their children [17,20,21,44]. In opposition, when the balance between work and family demands is perceived as positive and enriching, it is related to greater involvement and improved parental practices [16,17,45]. Importantly, aspects accounting for work conditions, such as lower number of hours at work, work stability, and flexibility, which are related to work–family gains, were associated with greater father involvement, namely in direct care activities and play [17,40,46,47,48,49].

Concerning personal influences on father involvement, parenting styles are addressed as one important indicator of the father–child relationship quality, potentially explaining lived experiences between fathers and their children [4,12]. Parenting styles reflect the emotional climate in which parents raise their children [11], conceptualizing how parents nurture their child and establish limit-setting, through parental responsiveness and demandingness, through three styles [50]: Authoritative parents balance demandingness and responsiveness by monitoring the child’s behavior, addressing clear limit standards. Authoritative parents are assertive in their interactions but not intrusive or restrictive. They offer supportive methods rather than punitive ones, appealing to self-responsibility, cooperation, and self-regulation. Otherwise, the authoritarian style is characterized by strict discipline and control. Authoritarian parents are demanding and directive but not responsive, relying on a punitive orientation. Finally, permissive parents are more responsive than demanding, avoiding confrontation in child interactions.

Overall, authoritative parenting has been related to more positive developmental outcomes in comparison with other ones [50,51,52,53]. Authoritative parenting has also been linked to higher father involvement with direct care [54,55]. Otherwise, authoritarian fathers tend to be less directly engaged with their children [52,56]. Despite the distinct characteristics of authoritarian and permissive styles regarding responsiveness and demandingness, they tend to be positively correlated and associated with negative parenting behaviors, being addressed as negative parenting styles [57,58].

Parenting styles, however, are influenced by contextual aspects, namely work–family demands. Work–family strains are related to greater control, more intrusive parenting, and harsh parental practices [18,59,60]. How work–family gains account for parental experiences has been much less explored but points to positive associations, namely due to the effort of working parents to better structure their available time to be with their children, investing in parenting [61]. Work–family gains are related to more positive parental practices and family satisfaction [17,18]. Despite the interconnection between those variables, there is a lack of studies examining the direct effects of ecological factors (i.e., work–family conflict) on father involvement and whether personal aspects, such as parenting styles, may mediate this association. This is relevant given that the literature points out the spillover effect of work–family balance on parental practices and roles [17,36]. Hence, it is important to examine both dimensions of work–family conflict to better understand how its dynamics may influence the way fathers are involved in their parenting.

The current study aims to explore, from the father’s perspective, whether parenting styles mediate the association between work–family gains/strains and father involvement in child direct and indirect care. We anticipate that work–family gains will be related to greater father involvement in both direct and indirect care through positive parenting styles. In opposition, we hypothesized that higher work–family strains would have detrimental consequences on father involvement in both dimensions of care through negative parental styles.

## 2. Materials and Methods

### 2.1. Participants

Fathers (*n =* 411) of children aged 21–72 months (*M* = 53.53; *SD* = 12.06) were invited to participate in our study both at public (39.2%) and private schools in the Centre and South regions of Portugal. Only working and married/cohabiting fathers were included in the sample. Over half of the children were firstborns (53.5%) and girls (55%). On average, fathers were 36.34 years old (*SD* = 6.15; range: 21–62 years old), reported a workload of 8.36 daily working hours (*SD* = 1.53; range 4–16), and, on average, had 11.06 years of education (*SD* = 3.82; range: 4–19 years of education).

### 2.2. Procedures

This study entails a larger project examining aspects of enhancing father involvement. The current study was presented in schools/kindergartens, and parents were invited to participate. Those who agreed received a letter explaining the study, a consent form, and a set of questionnaires assessing sociodemographic characteristics of the family, parental involvement, work–family conflict, and parental practices. Only working parents that were married/cohabiting were included in the current study. Previously, the study was approved by the Ethics Committee of the university (blind for review). All participants signed a consent form. The study was conducted in accordance with the Declaration of Helsinki and approved by the Institutional Review Board of [blind for review].

### 2.3. Materials

*Sociodemographic questionnaire.* This questionnaire was developed by the research team, aiming to characterize participants and their family backgrounds. Parents were categorized through a set of objective questions about aspects such as their age, education, marital status, working hours, family characteristics, e.g., place of residence, number of children, and their child, e.g., sex, age.

*Combining Work and Family Questionnaire* (Marshall & Barnett, 1993 [13]; Portuguese version Martins et al. 2008 [62]). Fathers answered a self-reported scale assessing two dimensions: (1) work–family gains (7 items), assessing the benefits of combining professional and parenting demands for their children and themselves (e.g., *Working helps me to better appreciate the time that I spend with my children*); and (2) work–family strains (13 items), evaluating the extent to which father’s employment-related constraints carry over from work to family duties (e.g., *Because of my work responsibilities my family time is less enjoyable and more pressured*), or vice versa (e.g., *Because of my family responsibilities, I have to turn down work activities or opportunities that I would prefer to take on*). Fathers answered on a 4-point Likert scale, with higher scores indicating more work–family conflict. In the current study, good internal consistency reliabilities were obtained for both Strain (α = 0.87) and Gains dimensions (α = 0.81). This scale has been widely used in previous research (e.g., [13,18,19]).

*Parental Involvement Scale: Caregiving and Socialization Activities* (Monteiro, Veríssimo, Pessoa, & Costa, 2008 [63]). Originally, it was a 26-item, self-reported questionnaire examining the father’s perception of his involvement, in relation to the mother, regarding child-related activities. It was answered on a 5-point scale (from “always the mother” to “always the father”), evaluating five dimensions: direct and indirect care, teaching/discipline, play, and outdoor leisure. In this study, we only included the subscale of direct and indirect care due to our main goal. Direct care assesses the direct involvement in the child’s daily activities of care, such as preparing meals and putting them to bed (e.g., *Who bathes the child*), whereas indirect care examines aspects related to taking decisions and responsibilities of activities related to the child’s well-being without direct interaction, such as scheduling doctor’s appointment, preparing meals (e.g., *Who usually buys your child’s clothes*). Acceptable internal consistency reliabilities were obtained for both direct (α = 0.73) and indirect care (α = 0.69). Several studies have used this measure (e.g., [34,44,55]).

*Parenting Styles and Dimensions Questionnaire* (Robinson et al., 2001; Portuguese version Pedro, Carapito, & Ribeiro, 2015 [64]). Fathers self-reported three dimensions of parenting according to Baumrind’s authoritative, authoritarian, and permissive typologies on a 5-point Likert scale. Higher scores in each sub-scale correspond to more frequent use of that parental style. The authoritative dimension corresponds to a positive parenting style, involving non-coercive discipline and responsiveness (e.g., *I encourage our child to talk about his/her problems*), whereas negative parenting refers to a style largely detrimental to children’s well-being, with recurrent punitive or coercive discipline, physical punishment, or involving authoritarian (e.g., *I scold and criticize our child to help him/her progress)* or permissive behaviors (e.g., *I avoid yelling and/or criticizing our child, even when s/he acts contrary to what is expected*). Given that the permissive and authoritarian styles are related to negative parenting practices (e.g., Braza et al., 2015; Williams et al., 2009), and they were correlated in our sample (*r* = 0.14, *p* < 0.001), these dimensions were merged in a negative parenting style. Good values of internal consistency were obtained: 0.88 for the positive style and 0.74 for the negative one. This scale has been widely used in previous studies (e.g., [50,51,52,53,54,55,56,57,58]).

### 2.4. Data Analysis

Data were analyzed using SPSS and AMOS (version 26; IBM, SPSS Inc., Chicago, IL, USA). Firstly, descriptive statistics were performed on all sociodemographic and study variables. Pearson correlations examined bivariate associations between sociodemographic variables, parental father involvement, parenting styles, and work–family conflict, namely, to identify possible covariates of the path model. Previously, the main analysis’s missing data patterns were examined. Given that none of the missing items were above 5% and were missing completely at random (Little’s MCAR tests > 0.05; [65]), missing data were imputed through Expectation Maximization. A preliminary descriptive analysis to examine means, variability, and Pearson correlations to explore simple associations among study variables was performed.

Structural equation modeling (SEM), using maximum-likelihood estimation, was used to examine the mediation role of parental styles (i.e., positive and negative) between the association of fathers’ gains and strains at work and their involvement with the child. SEM is a powerful multivariate technique that allows the capture of dynamic and complex relations between variables represented by arrows in path diagrams. With SEM, it is possible to examine how well a process model linking dependent variables (i.e., work–family strains and gains) to some outcomes (i.e., father involvement in direct and indirect care) through one or more intervening pathways (i.e., parenting styles) fits the observed data. SEM is helpful in the context of mediation analysis, namely by providing model fit information regarding the consistency of the hypothesized mediation model [65,66,67].

The quality of model adjustment was assessed using the following goodness-of-fit indices: the chi-square to degrees of freedom ratio (χ²/df; with values below 5 indicating good fit), the Bentler comparative fit index (CFI > 0.90), the goodness-of-fit index (GFI > 0.95), the standardized root mean square residual (SRMR < 0.06), and the root mean square error of approximation (RMSEA < 0.07; [66]). Finally, mediation was tested using bootstrap resampling procedures. Bias-corrected 95% confidence intervals (CI’s) for the unstandardized effects based on 5000 resamples were used in the bootstrap analysis [68].

## 3. Results

This section may be divided into subheadings. It should provide a concise and precise description of the experimental results, their interpretation, as well as the experimental conclusions that can be drawn.

### 3.1. Descriptive Analysis

Correlations between the study variables are displayed in Table 1. Overall, increased father work–family strains were related to lower involvement in indirect care (*r* = −0.18, *p* < 0.01), as well as with a lower positive style in parenting (*r* = −0.10, *p* < 0.05), whereas it was related to a higher negative style (*r* = 0.22, *p* < 0.01). Moreover, increased father work–family gains were related to both positive (*r* = 0.11, *p* < 0.05) and negative styles in parenting (*r* = 0.14, *p* < 0.05). Finally, a father’s direct involvement was related to a greater positive style (*r* = 0.13, *p* < 0.01). Neither parents’ education, age, number of working hours, nor child’s sex and age were simultaneously related to both dependent and independent variables (see Table 1 for details); thus, they were not included as co-variables in the subsequent analysis.

### 3.2. Mediation Analysis

**Figure 1:**
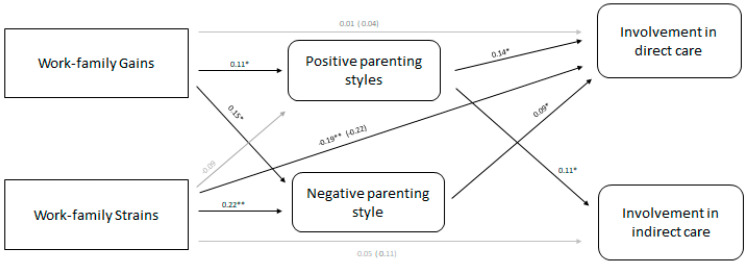
Path model representing the associations between work–family conflict, father involvement, and mediating role of parenting styles. Path values represent standardized regression coefficients. * *p* < 0.05; ** *p* < 0.01.

The model provided the following good fit indices: χ²(4) = 16.883; *p* = 0.002; χ^2^/*df* = 4.221; CFI = 0.933; GFI = 0.982; SRMR = 0.009; RMSEA = 0.042, *p*close = 0.392, 95% CI 0.000, 0.146). Figure 1 shows the significant standardized parameter estimates of the final mediational model.

Concerning significant direct effects, greater father work–family gains were positively related to positive parenting styles (*β* = 0.11, *p* < 0.05), and positive parental styles were positively related to both direct (*β* = 0.14, *p* < 0.01) and indirect care (*β* = 0.10, *p* < 0.05). Also, work–family gains were positively related to negative parenting styles (*β* = 0.15, *p* < 0.01). Concerning work–family strains, they were positively related to negative parental styles (*β* = 0.23, *p* < 0.001) and negatively related to father involvement with direct care activities (*β* = −0.19, *p* < 0.001). Results of the bootstrap analysis revealed that work–family gains had a positive indirect effect on greater involvement both in direct care and indirect care through positive parenting styles. Also, work–family strains had a negative indirect effect on indirect care through negative parenting styles (see Figure 1 and Table 2 for details).

Specifically, the analysis of indirect effects revealed that father work–family gains were positively associated with greater involvement in both direct and indirect care through positive parental styles. Work–family gains were also related to greater involvement in direct care through negative parenting styles. Otherwise, father work–family strains had an indirect effect on involvement in direct care through negative parenting styles.

## 4. Discussion

The current study examined, from fathers’ perspectives, how work–family gains and strains indirectly accounted for involvement with a child’s direct and indirect care through parenting styles. The striking finding of our study is that cumulative work and parenting demands per se were not detrimental to father involvement, being influenced by ecological and personal aspects. Fathers who perceived higher work–family gains reported greater involvement in both direct and indirect care through positive parenting styles. Otherwise, more work–family strains were related to involvement in direct care through negative parental styles.

Overall, these findings translated the variety of experiences on parenting, revealing how father involvement is influenced by ecological (i.e., experience of work–family demands) and personal (i.e., parenting styles) aspects. Despite previous studies having already identified these independent associations, e.g., [24,35], our study innovates by examining the mediating role of parenting styles. Possibly, by relying on positive parenting styles, fathers appeal to children’s self-responsibility and cooperation, which may facilitate their involvement with a child’s caregiving in direct activities, such as bathing or dressing. They may also feel more pleasure and with greater ability to broaden their scope of caregiving, being more involved with activities that do not require direct interactions—i.e., indirect care—but are crucial for a child’s well-being, such as preparing meals or buying clothes. The mediation of positive parenting styles, which involve flexible, supportive, and efficient approaches to managing professional and parental activities, may be reflected in a father’s sensitivity to solving parenting challenges, as well as to accommodate its multiple demands [18,69,70,71]. It is suggested that satisfaction and self-efficacy in the workplace are related to higher well-being, self-esteem, and positive emotions, which is conveyed to a more sensitive and warm parenting [16,18,46,49,69,71,72]. Indeed, work–family gains have been related to less irritable or hostile parenting [18,52], and fathers with positive parenting practices easily set positive limit standards, valuing children’s socio-emotional needs [50].

However, work–family gains were also related to father involvement in direct care through negative parenting styles. This unexpected result suggests that despite the pleasure related to parental and professional demands reflected in greater involvement with the child’s direct care, it happens through negative parenting styles characterized by negative forms of interaction, which may undermine the quality of father involvement. These associations may rely on some characteristics of the father’s professional role, such as greater control and monitoring, and mobilizing more authoritarian practices, reflected in their parenting styles [56,60].

Although the literature emphasizes how work–family strains limit parental involvement [12,14,17,18], this was not found in our study, contradicting our second hypothesis. Our findings revealed how father’s work–family strains were related to greater involvement in direct care activities through negative parenting styles. Indeed, fathers with more competing work–family demands tend to mobilize negative parenting practices, being over-reactive, mobilizing harsh discipline, and punitive and irritable responses to their child [18,56,60]. Possibly, despite their greater involvement in childcare, the ability to positively monitor child’s activities and to provide affectionate and responsive interactions is battered by their negative parenting styles [56,60,73]. On the one hand, fathers’ work–family strains have been linked to controlling parenting, as well as to less consistency in parental practices, with more irritability, hostility, and frustration in interactions [18,60,73], independent of child characteristics and the father’s mental health [18]. On the other hand, these fathers may hold more traditional perspectives, more tied to their role as the breadwinner, which may account for the greater difficulty of accommodating professional demands and working hours to family needs, increasing work–family conflict [71,74]. It may also be argued that greater work–family strains may limit the father’s ability to engage in the child’s non-routine activities, limiting the “parenting mood”, which may account for feeling distant from their child(ren), and less able to adequately interact with them, which may explain the mediation effect of the negative parenting styles [14]. Moreover, work–family strains may also generate a lack of physical and emotional energy, which may introduce negative consequences to the quality of interactions with the child. These fathers may wish to be involved in daily routines, but due to their exhaustion and overload, they were not able to do it through positive parenting practices [18,71]. This may be explained by the perspective that work–family strains decrease a father’s ability to persist in planned activities and to monitor and reinforce behavioral norms due their limited ability to sustain planned actions, as well as higher emotional and cognitive withdrawal [18,40], limiting their involvement with indirect care activities.

Findings highlight how positive experiences of conciliating professional and familiar activities are an important vehicle, not only to broaden involvement with childcare duties but also to foster a positive parenting experience. In opposition, the negative balance between work–family undermines parenting quality. Therefore, professional environments should adopt strategies to reduce work–family conflict, stimulating and contributing to a greater involvement of the fathers in childcare. Aspects such as lower working hours during a child’s early years and flexible work hours are related to lower work–family conflict and greater parenting quality [5,8] and should be adopted by employers.

### Limitations and Future Research

Despite the relevance of the findings, some limitations of the current study must be addressed. Firstly, data were cross-sectional, which limits the establishment of causal links between work–family conflict and father involvement. Future studies should examine how these variables evolve over time and their mutual influence, namely on sensitive periods of child development, such as transition to parenthood or return to work. Secondly, despite the value of assessing fathers’ perspectives, data relied on self-reported measures. Future studies should focus on how fathers really engage with their children, detailing not only their daily interactions but also the quality of these interactions [35]. Third, this study only examined work–family conflict on father involvement among employed and married fathers, and more complex working (e.g., unemployment) and familiar (e.g., divorce) situations should be explored. This is critical given that some studies have uncovered how vulnerable working conditions limit father involvement, namely among divorced fathers [75], which should be addressed by future research. Fourth, information about the specificity of work arrangements, such as shift work, professional roles, and flexible hours, is lacking, which may compromise a full understanding of its contribution to work–family conflict. Because work–family conflict has been highly related to working conditions, such as night work shifts or lower autonomy at work, e.g., [5,23], this should be better examined by future studies. Finally, given that the sample was relatively homogeneous concerning socioeconomic background and working hours, the understanding of the interplay of these variables on families from more impoverished and vulnerable contexts was limited, which should be addressed by future studies.

Despite previous research suggesting that longer working hours have been related to higher work–family conflict [5,18], this was not found in our study. Possibly, this is related to Portuguese working conditions, which are dominated by full-time jobs, decreasing the variability of parents’ working hours [22]. It would also be beneficial to explore other potential mediators that may explain the relationship between work strains/gains and father involvement. For example, factors such as work–life balance strategies or cultural norms could also play a role and should be considered in future research. Moderators of these associations, such as the level of social support or income, should also be examined.

Despite these limitations, our findings contribute to the state-of-the-art by exploring how work–family strains and gains in contemporary fatherhood spill over into parenting, affecting not only the dimensions in which fathers are involved with childcare but, more importantly, how they are involved through parenting practices. It is suggested that parenting quality is unfolded by the ability to compromise work and family demands, highlighting the critical role of the quality of parental practices [14,18,73]. Indeed, the balance of work and family activities may be positive when it happens through positive parenting styles, contributing to the family’s well-being. When work–family gains are perceived, it seems to promote greater involvement with caregiving tasks through positive parenting styles. In opposition, it is suggested that work–family strains may be detrimental to parenting by generating distress resulting in more adverse parental behaviors, reflected in a more restricted form of involvement. Future studies may examine how work–family conflict and parental styles account for other domains of parenting, such as the decrease of parental stress and burnout.

## Figures and Tables

**Table 1:** Pearson correlations and descriptive statistics for the main studied variables.

	M (SD); Range	1	2	3	4	5	6	7	8	9	10	11
1. WFS	1.81 (0.50); 1–4	-	-									
2. WFG	2.68 (0.56); 1–4	−0.05	-									
3. Direct care	2.44 (0.40); 1–3	−0.18 **	0.07	-								
4. Indirect care	2.81 (0.38); 1–4	−0.05	0.09	0.55 **	-							
5. Positive style	3.69 (0.53); 1–5	−0.10 *	0.11 *	0.13 **	0.09	-						
6. Negative style	2.06 (0.28); 1–3	0.22 **	0.14 *	0.02	0.03	−0.27 **	-					
7. Father’s age (years)	36.34; (6.15); 21–62	−0.09	0.09	−0.10 *	−0.03	0.04	−0.03	-				
8. Father’s education (years)	10.91 (4.16); 4–19	0.06	0.13 *	0.02	0.16 **	0.12 *	0.03	0.10 *	-			
9. Working hours		0.09	0.04	0.02	−0.05	0.04	−0.04	0.03	0.02	-		
10. Child’s sex (0 = girls; 1 = boys)	-	0.002	−0.01	−0.01	−0.14 *	0.07	−0.08	0.05	−0.03	0.04	-	
11. Child’s age (months)	53.53 (12.06); 20.66–72.90	−0.002	0.02	0.12 *	0.08	0.03	−0.11 *	0.08	−0.01	0.10 *	−0.04	-

* *p* < 0.05; ** *p* < 0.01.

**Table 2:** Indirect effects of work–family gains and strains on (in)direct dimensions of involvement.

	Estimate	*p*-Value	BC 95% CI Lower/Upper
Global indirect effects	Standardized		
WFG → Direct care	0.029	0.007 *	0.010/0.057
WFG → Indirect care	0.021	0.050 *	0.002/0.047
WFS → Direct care	0.020	0.669	−0.016/0.031
WFS → Indirect care	0.012	0.439	−0.016/0.028
Specific indirect effects	Unstandardized		
WFG → PPS → Direct care	0.011	0.028 *	0.002/0.027
WFG → PPS → Indirect care	0.007	0.050 *	0.001/0.022
WFG → NPS → Direct care	0.010	0.031 *	0.002/0.023
WFG → NPS → Indirect care	0.007	0.107	0.000/0.018
WFS → PPS → Direct care	−0.010	0.071	−0.030/-0.001
WFS → PPS → Indirect care	−0.007	0.088	−0.027/0.000
WFS → NPS → Direct care	0.017	0.037 *	0.003/0.035
WFS → NPS → Indirect care	0.011	0.140	−0.001/0.030

WFG: work–family gains; WFS: work–family strains; PPS: positive parenting styles; NPS: negative parenting styles. Standardized coefficients are presented for global indirect effects, and unstandardized coefficients are presented for specific indirect effects. * *p* < 0.05.

## Data Availability

Data are available under request to the corresponding author.
